# The Role of EZH2 in the Regulation of the Activity of Matrix Metalloproteinases in Prostate Cancer Cells

**DOI:** 10.1371/journal.pone.0030393

**Published:** 2012-01-17

**Authors:** Yong Jae Shin, Jeong-Ho Kim

**Affiliations:** Department of Biochemistry and Molecular Biology, The George Washington University Medical Center, Washington, D.C., United States of America; The University of Texas M.D Anderson Cancer Center, United States of America

## Abstract

Degradation of the extracellular matrix (ECM), a critical step in cancer metastasis, is determined by the balance between MMPs (matrix metalloproteinases) and their inhibitors TIMPs (tissue inhibitors of metalloproteinases). In cancer cells, this balance is shifted towards MMPs, promoting ECM degradation. Here, we show that EZH2 plays an active role in this process by repressing the expression of TIMP2 and TIMP3 in prostate cancer cells. The *TIMP* genes are derepressed by knockdown of EZH2 expression in human prostate cancer cells but repressed by overexpression of EZH2 in benign human prostate epithelial cells. EZH2 catalyzes H3K27 trimethylation and subsequent DNA methylation of the *TIMP* gene promoters. Overexpression of EZH2 confers an invasive phenotype on benign prostate epithelial cells; however, this phenotype is suppressed by cooverexpression of TIMP3. EZH2 knockdown markedly reduces the proteolytic activity of MMP-9, thereby decreasing the invasive activity of prostate cancer cells. These results suggest that the transcriptional repression of the *TIMP* genes by EZH2 may be a major mechanism to shift the MMPs/TIMPs balance in favor of MMP activity and thus to promote ECM degradation and subsequent invasion of prostate cancer cells.

## Introduction

Metastasis—the spread of cancer cells from a primary site to other parts of the body—is a common feature of malignant tumors. The process of cancer metastasis consists of multiple, sequential steps; cancer cells escape from the primary tumor, enter the bloodstream, travel to distant sites, and extravasate to form secondary tumor sites [1]. During metastasis, cancer cells invade and migrate through the normal molecular constraints, such as the extracellular matrix (ECM) [2]. The ECM, often referred to as the connective tissue, is an organized network of extracellular materials surrounding and supporting cells. The ECM is composed of a wide variety of polysaccharides and proteins, such as laminins, collagens, fibronectin, and proteoglycan, and plays an integral role in determining the shape, development, and biochemical function of cells [2]. The fabric of the ECM proteins makes up the basement membrane (BM) that underlies the basal surface of epithelial tissues and forms a physical barrier against tumor invasion. Cancer cells are capable of degrading the ECM barrier by using enzymes, resulting in dissolution of the BM. Most prominent among the enzymes are the matrix metalloproteinases (MMPs) [3].

MMPs are a large family of zinc-dependent endopeptidases and responsible for degradation of the ECM [4]. MMPs have long been known to be associated with physiological and pathological processes such as tissue remodeling, wound healing, angiogenesis, and cancer progression [5]–[8]. MMPs appear in latent proteins in the cytosol (pro-MMPs) and undergo proteolytic processing to yield the mature enzymes, which are, in turn, secreted and associated with the cell surface and the ECM [9], [10]. When displayed at the cell surface, however, MMPs are inhibited by the endogenous tissue inhibitors of metalloproteinases (TIMPs), which directly bind to the catalytic domains of MMPs in a 1∶1 stoichiometry [11]. Therefore, the balance between MMPs and TIMPs is critical for eventual ECM remodeling and degradation. The human genome encodes four TIMPs (TIMP1–TIMP4) that are functionally redundant and inhibit 23 human MMPs [12]. In many malignant tumors, expression of TIMPs is down-regulated, consistent with their role as MMP inhibitors [13], [14]. Suppression of TIMP expression by antisense RNA confers oncogenicity on Swiss 3T3 cells [15], [16]. Conversely, overexpression of TIMPs results in the inhibition of invasion and metastasis of cancer cells [17]–[23]. These observations indicate that repression of *TIMP* genes may be an important regulatory mechanism for cancer progression, but the underlying mechanism is still not fully understood.

EZH2 (polycomb group protein enhancer of zeste homolog 2) is the catalytic subunit of the polycomb repressive complex 2 (PRC2) [24], [25] and is overexpressed in a variety of human cancers [26]. Early studies showed that high levels of EZH2 expression are associated with invasion and metastasis of malignant tumors such as breast and prostate cancers [27]–[31] and that EZH2 overexpression transforms the benign prostate cells RWPE-1 [32] and BPH1 [33] and the immortalized breast epithelial cells [28]. Recently, EZH2 has also been found to regulate signaling pathways associated with cellular metabolism such as the Ras GTPase-activating protein DAB2IP [34], [35] and the adrenergic receptor-beta-2 ADRB2 [36], promoting cancer progression. EZH2 appears to mediate transcriptional silencing by either methylating lysine 27 in histone H3 (3meH3K27) [23], [24] or recruiting DNA methyltransferases (DNMTs) to its target genes that catalyze de novo DNA methylation [37]. However, recent reports have also shown that H3K27 trimethylation by EZH2 is not always associated with promoter DNA methylation for the silencing of certain EZH2-target genes [38]–[41]. The functional role of EZH2 in prostate cancer progression has been identified by gene expression profiling of RNA from nontumorigenic human prostate epithelial cells overexpressing EZH2 [27]. However, the results were found not to alter significantly expression levels of many metastasis–associated genes that were identified by genetic profiling of human prostate cancer cells [42]. The nature of this discrepancy is unclear but might arise from differences in expression levels of EZH2 in the prostate cancer cells tested.

In this study, we investigated the role of EZH2 in activation of MMPs to promote the invasion and metastasis of prostate cancer cells. To identify the metastasis-associated genes regulated by EZH2 in prostate cancer, mRNA expression in highly invasive prostate cancer cells in which EZH2 expression was knocked down was profiled using human metastasis PCR arrays. We found that high levels of EZH2 expression during cancer progression induce repression of *TIMP* genes (*TIMP2* and *TIMP3*), leading to increased activity of MMP-9 and thus to increased invasive activity of prostate cancer cells. These results provide for the first time evidence that EZH2 plays an active role in shifting the MMPs/TIMPs balance towards MMPs and thereby promoting metastasis of prostate cancer cells.

## Results

### EZH2 knockdown significantly reduces the invasive and migratory activities of prostate cancer cells

**Figure 1:**
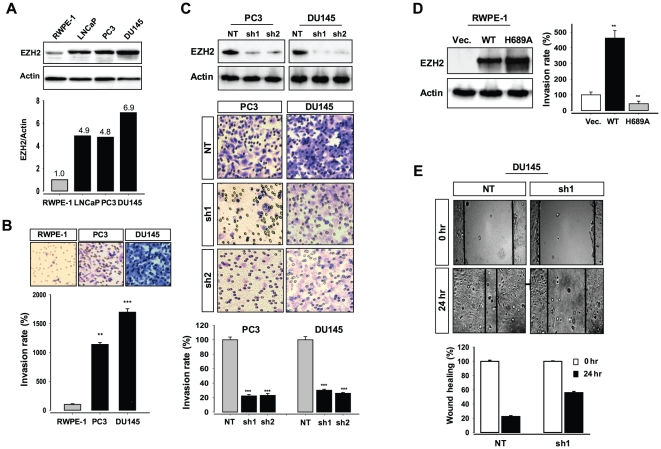
High levels of EZH2 promote invasion and migration of prostate cancer cells. (**A**) Western blot analysis of EZH2 expression in the benign prostate cell line RWPE-1 and the malignant prostate cancer cell lines LNCaP, DU145, and PC3 (top). Actin was used as an internal control. The protein bands were quantified using Quantity One software (Bio-Rad) (bottom). (**B**) Invasion assay of RWPE-1, PC3 and DU145 cells. Cell invasiveness was assessed by the invasion of cells through BME-coated inserts in the Transwell Boyden chamber (top). The invasion rate was determined by counting the cells that migrated through the inserts and expressed as the percentage relative to control (RWPE-1, set to 100%) (bottom). Each bar represents the mean ± s.e of five fields counted. (**C**) Western blot analysis of EZH2 expression in PC3 and DU145 cells after an infection with EZH2-specific shRNA lentivirus (sh1 or sh2; two different EZH2-specific shRNAs) or with non-treated shRNA (NT, control) lentivirus (top). Invasion assay of PC3 or DU145 cells after an infection with EZH2-specific shRNA (sh1 or sh2) or control (NT) lentivirus (middle). Representative fields of invaded and stained cells are shown (middle). Each bar represents the mean ± s.e of five fields counted (bottom). (**D**) Western blot analysis of overexpression of wild-type (WT) and mutant (H689A, enzymatically inactive) EZH2 proteins in RWPE-1 cells infected with EZH2 (WT)-GFP, EZH2 (H689A)-GFP, or control (GFP vector) lentivirus. EZH2 proteins were expressed from the CMV promoter (left). Invasion assay of RWPE-1 cells infected with EZH2 (WT)-GFP, EZH2 (H689A)-GFP, or control (GFP vector) lentivirus (right). (**E**) Wound healing assay of DU145 cells infected with EZH2-specific shRNA (sh1) or control shRNA (NT) lentivirus. Images were taken before (0 h) and after wound (24 h) (top). The results were expressed as the percentage of the remaining area determined by normalizing the area of wound after 24 h to the initial wound area at 0 h (set to 100%). Each bar represents the mean ± s.e of five fields measured (bottom).* P<0.05, ** p<0.005 *** p<0.001 (as compared with control values).

To investigate whether high levels of EZH2 expression is correlated with the invasive phenotype of prostate cancer cells, we first determined protein levels of EZH2 in human prostate cell lines (LNCaP, PC3, and DU145). Our Western blot analysis showed that EZH2 levels are significantly elevated in the cancer cell lines than in the benign human prostate epithelial cell line RWPE-1 (Figure 1A). We then examined the invasive activity of prostate cancer cells expressing different levels of EZH2 protein using Transwell Boyden chamber assay. To this end, EZH2 expression was knocked down using two different EZH2-specific shRNAs, sh1 or sh2 (Figure 1C, top). PC3 and DU145 cells were highly invasive, compared with the benign RWPE-1 cells (Figure 1B). However, EZH2 knockdown significantly decreased invasiveness of the prostate cancer cells (Figure 1C, middle); only 18∼20% of the prostate cancer cells infected with lentivirus expressing EZH2 targeting shRNA penetrated the BME membrane in the chamber (sh1 and sh2), compared with those cells infected with the control (NT, nontargeting shRNA) lentivirus (Figure 1C, bottom). On the contrary, overexpression of EZH2 conferred an invasive phenotype on RWPE-1 cells (Figure 1D, WT); however, RWPE-1 cells overexpressing a mutant EZH2 protein (EZH2-H689A) with significantly reduced HMT activity [44] did not display invasive activity (Figure 1D, H689A). Because EZH2 plays an active role in prostate cancer invasion (Figures 1C and 1D), we further examined the migratory activity of DU145 cells using the wound-healing migration assay (Figure 1E, top). We found that DU145 cells fill ∼80% and ∼50% of the wounded areas before (NT) and after (sh1) knockdown of EZH2 expression, respectively, at 24 h after scratching (Figure 1E, bottom). These results strongly suggest that high levels of EZH2 expression promote invasion and migration of prostate cancer cells.

### Identification of the downstream target genes of EZH2 associated with metastasis of prostate cancer

**Figure 2:**
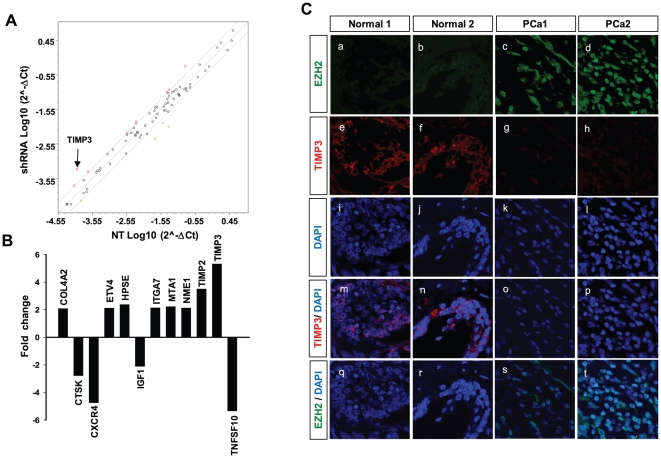
Identification of the downstream target genes of EZH2 associated with metastasis of prostate cancer. (**A**) RT^2^ profiler PCR array for Human Tumor Metastasis genes in DU145 cells after an infection with EZH2-specific shRNA or control (NT) lentivirus. The scatter plot of the test *vs* control samples indicates the validity of the experiment. (**B**) 12 genes whose expressions are highly affected by EZH2 knockdown are shown (also see Tables S1 and S2). Total RNA was isolated from DU145 cells after an infection with EZH2-specific shRNA or control (NT) lentivirus and processed for PCR array (**C**) Immunofluorescence images of normal prostate (Normal 1 and Normal 2) and prostate tumor tissue sections (PCa1 and PCa2), using anti-EZH2 antibody (green) and anti-TIMP3 antibody (red). Nuclei were stained with DAPI (blue).

To identify metastasis-associated genes whose expressions are regulated by EZH2, we investigated gene expression changes in invasive prostate cancer cells after EZH2 knockdown. Expression of different mRNAs in prostate cancer cells was assessed by using the human tumor metastasis RT^2^ *Profiler*™ PCR Array (PAHS-028D) designed to represent 84 genes known to be involved in metastasis (Figures 2A and 2B). The results showed that expression of 12 and 32 genes is altered >2-fold in DU145 (Table S1) PC3 (Figures S1A and S1B and Table S2) cells, respectively, by knockdown of EZH2 expression. Among those genes, TIMP3 was found to be the gene that is most highly upregulated in both DU145 and PC3 cells (Figures 2B and S1B), leading to the hypothesis that TIMP3 may be a primary target for repression by EZH2. To test this hypothesis, we examined protein levels of TIMP3 and EZH2 by immunohistochemistry using anti-EZH2 and anti-TIMP3 antibodies (Figure 2C). As shown in the immunostaining images, TIMP3 protein levels were high in normal prostate tissues (Figures 2C-e and 2C-f), but barely detectable in prostate cancer tissues (Figures 2C-g and 2C-h). Similar results were obtained by RT-PCR analysis showing that TIMP3 mRNA levels are markedly decreased in PC3 and DU145 cells, compared with the RWPE-1 cells (Figure S1C). In contrast, EZH2 protein levels were very high in prostate cancer tissues (Figures 2C-c and 2C-d) compared with normal prostate tissues (Figures 2C-a and 2C-b). These observations suggest that expression of TIMP3 and EZH2 is inversely correlated, probably due to downregulation of TIMP3 expression by EZH2.

### Downregulation of TIMP3 expression by EZH2 in prostate cancer cell lines

**Figure 3:**
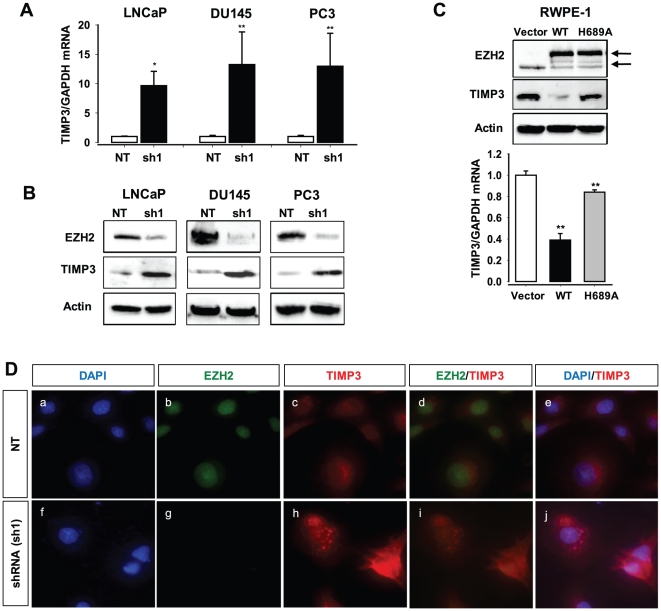
Expression of TIMP3 is downregulated by EZH2 in prostate cancer cells. (**A**) qRT–PCR analysis of expression of TIMP3 in the prostate cancer cells (LNCaP, DU145, and PC3) after an infection with EZH2-specific shRNA (sh1) or control shRNA lentivirus (NT). * P<0.05, ** p<0.005 (as compared with control (NT)). (**B**) Western blot analysis of endogenous levels of EZH2 and TIMP3 protein in prostate cancer cells (LNCaP, DU145, and PC3) infected with EZH2-specific shRNA (sh1) or control shRNA virus (NT). Actin was used as an internal control. (**C**) Western blot (top) and qRT–PCR (bottom) analyses of expression of EZH2 and TIMP3 in the benign prostate RWPE-1 cells after an infection with EZH2 (WT)-GFP, EZH2 (H689A)-GFP, or control (GFP vector) lentivirus. Actin was used as an internal control. * P<0.05, ** p<0.005 (as compared with control (vector)). The solid arrow indicates EZH2-GFP expressed from the CMV promoter, the dotted arrow indicates endogenous EZH2. (**D**) Immunostaining of EZH2 and TIMP3 expressed in DU145 cells infected with EZH2-specific shRNA (sh1) or control (NT) lentivirus, using anti-EZH2 antibody (green) and anti-TIMP3 antibody (red). Nuclei were stained with DAPI (blue). The merged images were also shown (EZH2/TIMP3 and DAPI/TIMP3).

To investigate further the transcriptional repression of TIMP3 expression by EZH2, we quantitatively determined TIMP3 mRNA and protein levels in three prostate cancer cell lines (LNCaP, PC3, and DU145) with and without knockdown of EZH2 expression. TIMP3 mRNA levels were found to be ∼10–13-fold higher in the cancer cells treated with EZH2-specific shRNA (sh1) than control cells untreated with the shRNA (Figure 3A), and as a result, TIMP3 protein levels were significantly increased by knockdown of EZH2 expression (Figure 3B). In contrast, overexpression of the functional EZH2 (WT), but not of the mutant EZH2 protein (H689A), was shown to decrease TIMP3 mRNA and protein levels in RWPE-1 cells (Figure 3C). Down-regulation of TIMP3 expression by EZH2 was also confirmed by immunostaining of TIMP3 in DU145 cells infected with EZH2-specific shRNA lentivirus (sh1) or control (nontargeting shRNA) lentivirus (NT) (Figure 3D). TIMP3 protein levels were low in DU145 cells (Figure 3D-c) but significantly elevated by knockdown of EZH2 expression (Figure 3D-h). Thus, TIMP3 may be a target of EZH2 for repression in prostate cancer cells.

### Epigenetic silencing of TIMP3 expression by EZH2

**Figure 4:**
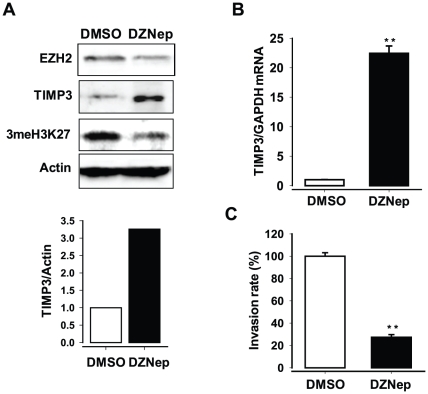
Repression of TIMP3 expression by histone methylation (3meH3K27) in prostate cancer cells. (**A**) Western blot analysis of endogenous levels of EZH2, TIMP3 and 3meH3K37 in the prostate cancer cells (PC3 cell) after treatment with DZNep (5 µM) or DMSO (control) for 2 days. Actin was used as an internal control. (**B**) qRT–PCR analysis of expression of TIMP3 in PC3 cells treated with DZNep (5 µM) or DMSO (control). (**C**) Invasion assay of PC3 cells treated with DZNep (5 µM) or DMSO (control).

EZH2 is a component of PRC2 that catalyzes trimethylation of H3K27 (3meH3K27) [24], [25]. To explore the underlying mechanism of the epigenetic repression of TIPM3 by EZH2, we investigated effects of the histone methylation inhibitor 3-Deaza-naplanocin A (DZNep) on expression of TIMP3. DZNep treatment resulted in decreased levels of TIMP3 protein (Figure 4A), probably by causing derepression of TIMP3 expression (Figure 4B). Moreover, the invasive potential of PC3 cells was ∼5-fold decreased by DZNep treatment (Figure 4C).

**Figure 5:**
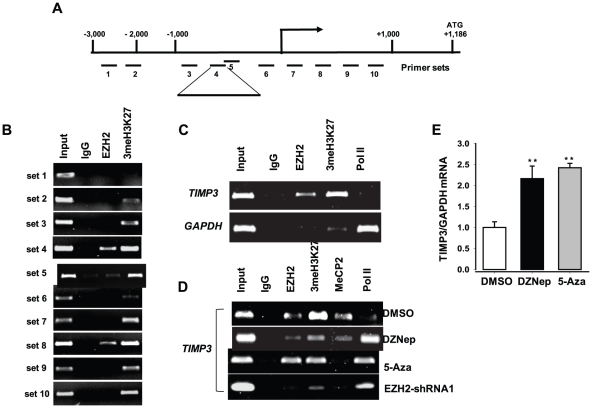
Transcriptional silencing of TIMP3 by EZH2. (**A**) Schematic representation of the promoter region of the *TIMP3* gene. The bent arrow represents the transcription start sites (+1). The lines below the *TIMP3* locus represent the regions amplified by PCR. (**B–C**) Immunoprecipitated DNA was analyzed by PCR with specific primer sets (also see Table S3). Chromatin obtained from PC3 (B) and DU145 (C) cells were immunoprecipitated using antibodies to EZH2, trimethyl-histone H3K27 (3meH3K27), RNA Polymerase II (Pol II), and IgG. The immunoprecipitated DNA was analyzed by PCR with the primer sets to amplify the region 4 in Figure 5A. The *GAPDH* promoter was used as a negative control. Each ChIP experiment was repeated at least three times and a representative experiment is shown. (**D**) Epigenetic regulation of TIMP3 expression in PC3 cells. ChIP assay was carried out using the antibodies to EZH2, 3meH3K27, MeCP2, RNAP II (Pol II), and IgG control after treatment with DZNep (1 µM), 5-Aza (10 µM), DMSO (control), or EZH2-specific shRNA (shRNA1). The immunoprecipitated DNA was analyzed by PCR with the primer sets to amplify the region 4 in Figure 5A. (**E**) qRT–PCR analysis of expression of TIMP3 in PC3 cells after treatment with DZNep (1 µM), 5-Aza (10 µM), or DMSO (control) for 2 days. The experiments were repeated at least twice and results were expressed as a ratio of TIMP2 or TIMP3 mRNA to GAPDH mRNA control. * P<0.05, ** p<0.005 (as compared with control (DMSO)).

To study further the molecular mechanism of the transcriptional repression of TIMP3 expression by EZH2, we performed ChIP assays in DU145 and PC3 cells using anti-EZH2 and anti-3meH3K27 antibodies. Because PcG proteins are recruited to DNA via the DNA-binding protein YY1 [45], the immunoprecipitated DNA was analyzed by PCR with the primer sets designed to amplify regions containing the YY1-binding sites in the *TIMP3* promoter (Figure 5A). The results indicate that: 1) EZH2 binds to the *TIMP3* promoter and catalyzes trimethylation of H3K27 (3meH3K27) (Figures 5B and 5C), but does not bind to the *GAPDH* promoter, served as a negative control (Figure 5C); 2) RNA polymerase II (RNAP II) binds strongly to the *GAPDH* promoter but has no appreciable binding to the *TIMP3* promoter (Figure 5C). These results suggest that EZH2-mediated trimethylation of H3K27 prevents RNAP II from binding to the *TIMP3* promoter and thereby results in transcriptional silencing of the gene.

We next determined whether H3K27 trimethylation is coupled with promoter DNA methylation for the repression of TIMP3, we treated PC3 cells with DZNep or the DNA methylation inhibitor 5-Aza followed by ChIP analysis. EZH2 knockdown or DZNep treatment markedly inhibited both H3K27 trimethylation and DNA methylation (MeCP2, methyl CpG binding protein 2) of the *TIMP3* promoter; in contrast, 5-Aza treatment inhibited only DNA methylation but had no significant effect on H3K27 trimethylation of the promoter (Figure 5D). Furthermore, treatment of PC3 cells with DZNep or 5-Aza resulted in derepression of TIMP3 expression to almost the same degree (Figure 5E). Thus, these results suggest that transcriptional repression of the *TIMP3* gene occurs through the EZH2-mediated H3K27 trimethylation and subsequent DNA methylation.

### Epigenetic silencing of TIMP2 expression by EZH2

**Figure 6:**
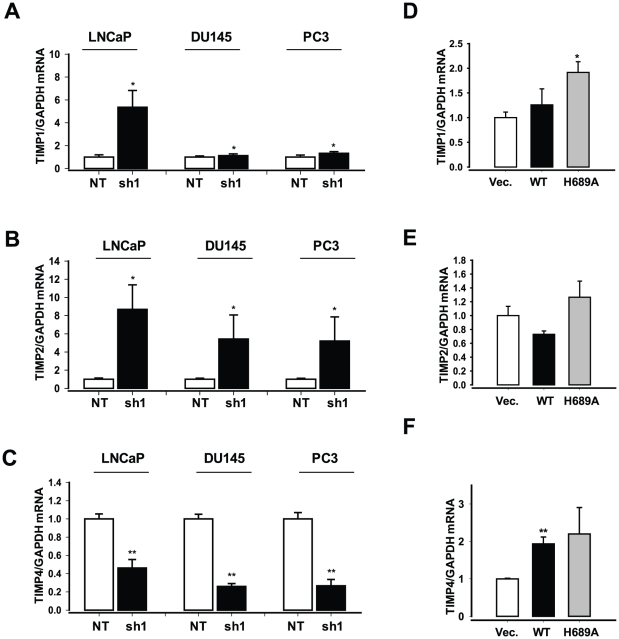
Expression of TIMPs is differently regulated by EZH2. (**A–C**) qRT–PCR analysis of expression of *TIMP* genes (A, TIMP1; B, TIMP2; C, TIMP4) in the prostate cancer cells (LNCaP, DU145, and PC3) after an infection with EZH2-specific shRNA (sh1) or control shRNA lentivirus (NT). * P<0.05, ** p<0.005 (as compared with control (NT)). (**D–F**) qRT–PCR analysis of expression of *TIMP* genes (D, TIMP1; E, TIMP2; F, TIMP4) in the benign prostate cell line RWPE-1 after an infection with EZH2 overexpression lentivirus (WT or H689A) or control virus for 4 days. Results were expressed as a ratio of *TIMP* mRNAs to *GAPDH* mRNA control. * P<0.05, ** p<0.005 (compared with control (GFP)).

**Figure 7:**
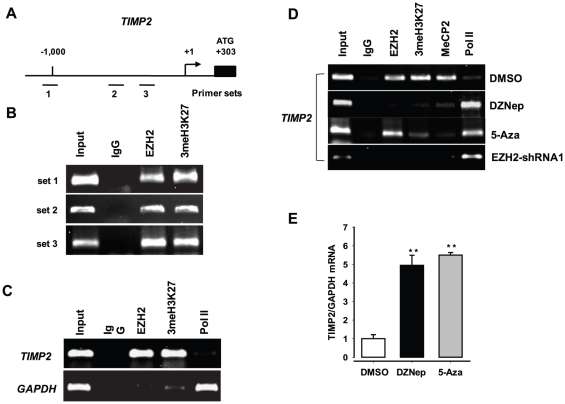
Transcriptional silencing of TIMP2 by EZH2. (**A**) Schematic representation of the promoter regions of the *TIMP2* gene. The bent arrow represents the transcription start sites (+1). The lines below the *TIMP2* locus represent the regions amplified by PCR using primer sets (set 1, set 2, and set 3 in (B); set 2 in (C) and (D)) designed to amplify regions containing the YY1-binding sites (see Table S3). (**B–C**) Chromatin immunoprecipitation (ChIP) experiments were performed with chromatin isolated from PC3 (B) and DC145(C) cells using antibodies to EZH2, 3meH3K27, and IgG. Results of our ChIP in both PC3 and DU145 cells are very similar to each other. Shown are ChIP results in DU145 cells. (**D**) Epigenetic regulation of TIMP2 expression in PC3 cells. ChIP assay was carried out using the antibodies to EZH2, 3meH3K27, MeCP2, RNAP II (Pol II), and IgG control after treatment with DZNep (1 µM), 5-Aza (10 µM), DMSO (control), or EZH2-specific shRNA (shRNA1). (**E**) qRT–PCR analysis of expression of TIMP2 in PC3 cells after treatment with DZNep (1 µM), 5-Aza (10 µM), or DMSO (control) for 2 days. The experiments were repeated at least twice and results were expressed as a ratio of *TIMP2* mRNA to *GAPDH* mRNA control. * P<0.05, ** p<0.005 (as compared with control (DMSO)).

We next investigated whether, like TIMP3, other three TIMPs (TIMP1, TIMP2 and TIMP4) are transcriptionally repressed by EZH2. Our RT-PCR analysis showed that TIMP1 was not regulated by EZH2, whereas TIMP4 was repressed when EZH2 expression is knocked down (Figure 6). In contrast, TIMP2 expression was significantly increased by EZH2 knockdown, but was not increased when the mutant EZH2 protein (EZH2-H689A) is expressed. Our ChIP studies show that TIMP2, like TIMP3, is also subject to EZH2-mediated histone methylation and subsequent promoter DNA methylation (Figure 7). Down-regulation of the *TIMP2* gene in prostate cancer cells was shown to be associated with promoter methylation [13], suggesting that epigenetic regulatory factors, such as HDAC and DNMT, may be involved in regulation of TIMP2. Our results show that the epigenetic modifier EZH2 may be the factor that mediates epigenetic inactivation of TIMP2.

### Transcriptional repression of TIMP3 by EZH2 promotes invasion of prostate cancer cells

**Figure 8:**
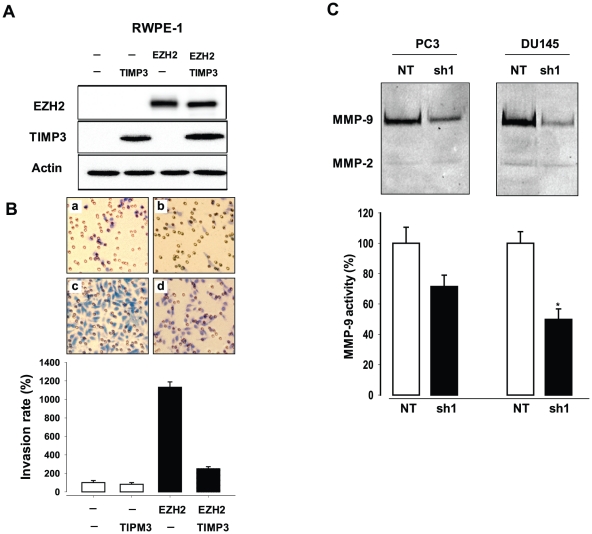
Transcriptional repression of TIMP3 by EZH2 promotes invasion of prostate cancer cells. (**A**) Western blot analysis of EZH2 and TIMP3 expression in RWPE-1 cells after an infection with the indicated combinations of lentivirus expressing EZH2, TIMP3 and control virus. (**B**) Invasion assay of RWPE-1 cells after an infection with the indicated combinations of lentivirus expressing EZH2, TIMP3 and control virus. Top: a, EZH2(−)/TIMP3(−); b, EZH2(−)/TIMP3(+); c, EZH2(+)/TIMP3(−); d, EZH2(+)/TIMP3(+). (**C**) Gelatin zymographic assay for MMP-2 and MMP-9 activity in PC3 and DU145 cells infected with EZH2-specific shRNA (sh1) or control shRNA virus (NT). The upper and lower bands indicate active MMP-9 (92 kDa) and active MMP-2 (62 kDa), respectively. Zymographic band intensities were quantified by densitometry using Quantity One software (Bio-Rad). Each bar represents the mean ± s.e of three fields counted. * P<0.05, ** p<0.005, *** p<0.001 (as compared with control (NT)).

To assess the functional link between the downregulation of TIMP3 by EZH2 (Figure 3) and the decreased invasive phenotype of prostate cancer cells by EZH2 knockdown (Figures 1C and 1D), we overexpressed EZH2 and TIMP3 separately or together in RWPE-1 cells (Figure 8A) and examined the invasive activity of the cells using Transwell Boyden chamber assay (Figure 8B). The invasive phenotype of RWPE-1 cells was induced by EZH2 overexpression as observed above (Figure 1D), but it was significantly suppressed by cooverexpression of TIMP3 (Figure 8B), providing direct evidence that the repression of TIMP3 by EZH2 results in increased invasive activity of prostate cancer cells.

MMP-2 and MMP-9 are known to be the prominent MMPs responsible for ECM degradation, and thus we tested whether enzymatic activity of these two MMPs is regulated by EZH2 using gelatin zymography assay. MMP-2 activity was found not to be changed significantly by EZH2 knockdown. However, MMP-9 activity was decreased by ∼28% in PC3 cells and ∼50% in DU145 cells, respectively, treated with EZH2-specific shRNA, compared with control cells (NT) (Figure 8C). Taken together, these results suggest that EZH2-mediated transcriptional repression of TIMP3 directly leads to activation of MMP-9.

## Discussion

The balance between MMPs and TIMPs is a critical parameter for the degradation of ECM and thus a crucial step for cancer invasion and metastasis. In this study, we show that aberrant upregulation of EZH2 expression in prostate cancer cells shift this balance towards MMPs and thereby promote degradation of the ECM. EZH2 does this by downregulating expression of *TIMP* genes (*TIMP2* and *TIMP3*). TIMP2 and TIMP3, compared with TIMP1 and TIMP4, inhibit a broad spectrum of MMPs and several of disintegrin-metalloproteinases, ADAMs and ADAMTSs [12]. Furthermore, TIMP3 is the only member of the TIMP family that tightly binds to ECM and thus is implicated in disease pathogenesis [9], [10]. TIMP3 also has an inhibitory effect on angiogenesis by blockage of VEGF binding to VEGF receptor-2 [46] and an ability to promote apoptosis [47]–[49]. Therefore, the transcriptional repression of TIMP2 and TIMP3 by EZH2 likely enhances ECM degradation and angiogenesis but reduces apoptotic activity, favoring cancer cell invasion and metastasis.

The presence or elevated expression of some of MMPs is positively associated with cancer progression [3], [50]. Our metastasis PCR array analysis shows that some *MMP* genes, such as *MMP7* and *MMP13*, in PC3 cells were markedly downregulated by knockdown of EZH2 expression (Figure S1). Thus, EZH2 seems to act as an activator, rather than a repressor, of the expression of those genes. However, these results were not clearly observed in DU145 cells, perhaps due to different genetic backgrounds between the two cell lines [51]. While the transcriptional regulation of MMP genes by EZH2, which was out of scope of this study, is currently under investigation, our results support the possibility that overexpression of EZH2 in metastatic prostate cancer cells may elevate overall MMP activity both by decreasing the levels of TIMPs (TIMP2 and TIMP3) and by increasing the levels of some MMPs, such as MMP-9.

Promoter DNA methylation is the most common epigenetic modification associated with gene silencing in cancer. EZH2 appears to recruit DNMTs to its target genes, resulting in methylation of adjacent CpG islands and subsequent gene silencing [37]. In this model, EZH2-mediated H3K27 methylation may be a prerequisite for promoter DNA methylation. Our results support this idea, as we have observed that *TIMP2* and *TIMP3*, like other EZH2 target genes such as *MSMB* [31], *RUNX3* [52], and *SLIT2* [39], are silenced through histone (H3K27) methylation followed by DNA methylation (Figures 5 and 7). However, promoter DNA methylation is not always required for repression of the EZH2 target genes. For example, EZH2-mediated silencing of the E-cadherin gene occurs through H3K27 methylation but does not require promoter DNA methylation [38]. In addition, H3K27 trimethylation is not associated with promoter DNA methylation for silencing of a substantial number of EZH2 target genes in prostate cancer [41]. Therefore, further studies are required to distinguish these mechanisms.

In order for cancer cells to metastasize, they must break free from the normal physical barriers, to migration and invasion, such as ECM and cell-cell adhesion. Moreover, degradation of the ECM releases signaling molecules associated with the ECM, such as growth factors and cytokines, that significantly influence metastasis [53]. Thus, the ECM degradation by MMPs is of pivotal importance for not only eliminating the barriers but also making the signaling molecules accessible to cancer cells. As demonstrated in this study, EZH2, which acts as a potential oncogene in various malignancies, plays an active role in the upregulation of MMP activity and promote ECM degradation and subsequent invasion of prostate cancer cells, providing a novel insight into the role of EZH2 in prostate cancer metastasis.

## Materials and Methods

### Cell culture

All cell lines used in this study were obtained from the American Type Culture Collection (ATCC; Rockville, MD). PC3, DU145 and LNCaP cells were cultured in RPMI 1640 with 10% FBS and penicillin/streptomycin at 37°C in a humidified atmosphere of 5% CO_2_. The RWPE-1 cells were cultured in keratinocyte serum-free medium (K-SFM) containing 50 µg/ml bovine pituitary extract and 5 ng/ml epidermal growth factor as described previously [43]. The cells were fed with fresh growth medium every 3 days, and at confluence, they were subcultured at a 1∶4 ratio using trypsin-EDTA (0.05%).

### Lentiviral infection

Lentiviral transduction was used for EZH2 knockdown and EZH2 overexpression in prostate cancer cells. Lentiviral expression vectors for GFP (green fluorescent protein; as a control), wild-type EZH2-GFP, and mutant EZH2 (H689A)-GFP were constructed by subcloning corresponding cDNAs into pLenti4/V5-DEST vector (Invitrogen). We obtained lentiviral short hairpin RNAs (shRNAs) specific for EZH2 from Sigma-Aldrich (MISSION® shRNA). Biologically active shRNAs were generated from the pLKO.1-puro vector, utilizing the Polymerase III U6-RNA promoter. The shRNA sequences for EZH2 knockdown were 5′CCGGTATGATGGTTAACG GTGATCACTGAGTGATCACCGTTAACCATCATATTTTTG-3′ (TRCN18365, sh1) and 5′-CCGGGCTAGGTTAATTGGGACCAAACTCGAGTTTGGTCCCAATTAACCTAGCTTTTTG-3′ (TRCN40074, sh2). Because these two shRNAs are equally effective in suppression of *EZH2* mRNA expression, we used EZH2-specific shRNA1 (sh1) in some of our experiments. For viral production, 293T cells were cotransfected with pLKO.1, pLenti4-EZH2-WT, pLenti4-EZH2-H689A or pLKO.1-EZH2-specific shRNA, and packaging plasmids (psPAX2 and pCMV-VSV-G) using CalPhos Mammalian Transfection Kit (Clontech). The virus-containing supernatants were collected 48–72 h after transfection and aliquoted to be stored at −80°C.

### Quantitative real-time PCR

Expression of *TIMP* and *GAPDH* mRNA was measured by real-time quantitative PCR using SYBR Green PCR Mastermix (Quanta biosciences). Total RNA was isolated from prostate cancer cells using Trizol Reagent (Invitrogen). 2 µg of total RNA was used for reverse transcription reactions using qScript cDNA SuperMix Kit following the manufacturer's instructions (Quanta biosciences). Results shown were a representative of three independent experiments. The primers used in this study are listed in Table S3.

### Western blot analysis

Cells were lysed in RIPA buffer (50 mM Tris pH 7.4, 150 mM NaCl, 1% NP-40, 0.5% Sodium deoxycholate, 0.1% SDS) supplemented with protease inhibitor cocktail (Roche Applied Science) or lysed directly in 2× SDS-PAGE sample buffer, and subjected to Western blot analysis using antibodies against EZH2 (BD Biosciences, 612666), TIMP2 (Millipore Inc., MAB3310), TIMP3 (Abcam, ab39184), 3meH3K27 (Millipore Inc., 07-449), MeCP2 (Millipore Inc., 07-013), RNA polyersase II (Millipore Inc., 05-623), and Actin (Sigma-Aldrich, A2066). Protein bands were visualized using West Pico chemiluminescent detection system (Pierce) and subjected to densitometry analysis using the ChemiDoc XRS system with Quantity One software (Bio-Rad Laboratories).

### Immunostaining for EZH2 and TIMP3

4–5-µm-thick, formalin-fixed, paraffin-embedded human prostate cancer and normal sections were purchased from Cybrd and BioChain Institute, Inc. Prostate cancer cells were grown on chamber slides (Nunc) and infected with either control or EZH2-specific shRNA lentivirus. After cultivating for 2–4 days, the cells were fixed with cold methanol and permeabilized with buffer containing 1% BSA, 10% normal goat serum, 0.3 M glycine, and 0.1% PBS-Tween for 1 h. The resulting cells were incubated with anti-TIMP3 and anti-EZH2 antibodies at 4°C overnight. After washing, they were incubated with Alexa Fluor® 594 goat anti-rabbit IgG and Alexa Fluor® 488 goat anti-mouse IgG at a 1/1000 dilution for 1 h. After mounting with mounting medium containing DAPI (Vector Labs, H-1200), the cells were photographed using a fluorescence microscope (Olympus Imaging America Inc, IX71 microscope).

### Cell invasion and migration assays

Cell invasion assay was performed using the cell invasion kit (Transwell Boyden's chamber with Transwell® Permeable Support Inserts Coated with Cultrex® BME (basement membrane extract), Corning Costar) according to the manufacture's instruction. In brief, serum-starved RWPE-1, DU145, or PC3 cells (2×10^5^ cells/well) were added to the upper chamber, whereas RPMI 1640 medium containing 10% fetal bovine serum was added to the lower chamber and incubated in 5% CO_2_ at 37°C for 24 h. Nonmigrating cells were gently removed from the upper surface of the filter with a cotton swab; migrating cells attached on the lower surface of the filter were fixed and stained with hematoxylin for counting. For the wound-healing migration assay, DU145 cells were seeded at 70% confluence into 6-well culture dishes and, 24 hours later, infected with EZH2-specific or control shRNA lentivirus. After the cells grew to confluence, scratch wounds were made on the confluent monolayers using sterile pipette tips. Afterward, the culture medium was replaced with fresh, complete medium and cells were incubated at 37°C for 24 h and then fixed and photographed. Cell migration was monitored using Olympus IX71 microscope, cell-migration areas were calculated with DP2-BSW application software (Olympus Imaging America Inc.).

### Chromatin immunoprecipitation (ChIP) assay

Chromatin was isolated from prostate cancer cell lines using the ChIP assay kit (Milipore Inc.) and precipitated with anti-EZH2, anti-3meH3K27, anti-MeCP2, or anti-RNA polymerase II antibodies. Immunoprecipitated DNA was PCR-amplified with the primer sets covering specific regions of the *TIMP2* and *TIMP3* promoters (Table S3). To amplify the DNA within a linear range of amplification for each gene, PCR was performed with different number of cycles or with a serial dilution of input DNA, and all results shown fall within this range. PCR products were run on agarose gels and visualized by ethidium bromide staining.

### Zymography assay

Gelatin zymography assay was performed using confluent prostate cells, which were maintained in serum-free media for 24 h and concentrated using an Amicon Ultra 10 k (Millipore). Equal amounts of concentrated media were mixed with the SDS sample buffer which did not contain β-mercaptoethanol and loaded onto SDS-PAGE gels containing 0.2% gelatin (Sigma). After electrophoresis, gels were washed three times for 20 min at room temperature with renaturing buffer (2.5% Triton X-100). They were then equilibrated in developing buffer (50 mM Tris-HCl pH 7.5, 0.15 M NaCl, 10 mM CaCl_2_ and 0.05% NaN_3_) at room temperature with gentle agitation for 20 min. After removing the developing buffer, the gels were incubated with fresh developing buffer at 37°C for 24 h. Finally, the gels were washed with distilled water, stained with 0.05% Coomassie Brilliant Blue for 1 h, and destained with destaining solution (45% methanol and 10% acetic acid) for 3 h.

### Chemicals

The anti-actin antibody (A2066), 5-Aza-2′-deoxycytidine (5-Aza, A3656), and EZH2 specific-shRNAs were obtained from Sigma-Aldrich. The anti-TIMP2 (MAB3310), anti-3meH3K27 (07-449), anti-MeCP2 (07-013), anti-RNA polymersase II (05-623) antibodies were purchased from Millipore. The anti-EZH2 antibody (612666) was obtained from BD Biosciences. 3-Deazaneplanocin A (DZNep, 13828) was purchased from Cayman. Anti-TIMP3 (ab39184) was obtained from Abcam.

## Supporting Information

Figure S1 **Identification of the downstream target genes of EZH2 associated with metastasis of prostate cancer.** (**A**) RT^2^ profiler PCR array for Human Tumor Metastasis genes in PC3 cells after an infection with EZH2-specific shRNA or control (NT) lentivirus. The scatter plot of the test *vs* control samples indicates the validity of the experiment. (**B**) 21 genes whose expressions are highly affected by EZH2 knockdown are shown (also see Tables S1 and S2). Total RNA was isolated from PC3 cells after an infection with EZH2-specific shRNA or control (NT) lentivirus and processed for PCR array. (**C**) qRT-PCR analysis to determine endogenous levels of *TIMP3* mRNA in the benign prostate cell line RWPE-1 and the malignant prostate cancer cell lines PC3 and DU145. Results were expressed as a ratio of the *TIMP3* mRNA to the house keeping gene *GAPDH* (glyceraldehyde-3-phosphate dehydrogenase) mRNA control. * P<0.05, ** p<0.005 *** p<0.001 (as compared with control values).
(EPS)

Table S1 **List of genes differentially expressed in DU145 cells after EZH2 knockdown using a Human Tumor Metastasis Real-time PCR Array.**
(DOC)

Table S2 **List of genes differentially expressed in PC3 cells after EZH2 knockdown using a Human Tumor Metastasis Real-time PCR Array.**
(DOC)

Table S3 **Primers for RT-PCR and ChIP analyses.**
(DOC)
