# Supplementary material for: The Role of EZH2 in the Regulation of the Activity of Matrix Metalloproteinases in Prostate Cancer Cells
Source: PLoS One. 2012 Jan 17;7(1):e30393. doi: 10.1371/journal.pone.0030393 (PMC3260297; doi:10.1371/journal.pone.0030393)
Supplement: Table S1 — List of genes differentially expressed in DU145 cells after EZH2 knockdown using a Human Tumor Metastasis Real-time PCR Array. (DOC) [file pone.0030393.s002.doc]

**Table S1. List of genes differentially expressed in DU145 cells after EZH2 knockdown using a Human Tumor Metastasis Real-time PCR Array**

| ***Gene*** | | | ***Fold change*** | ***Location*** | ***Function*** |
| --- | --- | --- | --- | --- | --- |
| **Upregulated genes** | | | |  |  |
| **CD44** | | +1.00 | | 11p13 | cell adhesion and stroma attachment |
| **COL4A2** | | +2.08 | | 13q34 | the component of extracellular matrix |
| **CST7** | | +1.15 | | 20p11.21 | inhibits cysteine proteinase |
| **CTBP1** | | +1.38 | | 4p16 | inhibits cell proliferation |
| **CTSL1** | | +1.48 | | 9q21-q22 | protein hydrolysate |
| **ETV4** | | +2.11 | | 17q21 | transcription factor, promotes proliferation |
| **FGFR4** | | +1.84 | | 5q35.1 | promotes invasion |
| **FLT4** | | +1.48 | | 17q21 | transcription factor, promotes proliferation |
| **FN1** | | +1.03 | | 2q34 | participates in cell adhesion |
| **FXYD5** | | +1.33 | | 19q12-q13.1 | negative regulation of cell adhesion |
| **KISS1R** | | +1.06 | | 19p13.3 | suppresses metastasis |
| **HPSE** | | +2.37 | | 4q21.3 | hydrolyses protein |
| **IL18** | | +1.42 | | 11q22.2-q22.3 | promotes cell proliferation |
| **ITGA7** | | +2.13 | | 12q13 | participates in cell adhesion |
| **ITGB3** | | +2.00 | | 17q21.32 | participates in cell adhesion |
| **CD82** | | +1.58 | | 11p11.2 | metastasis suppressor |
| **KISS1** | | +1.04 | | 1q32 | suppresses metastasis |
| **RPSA** | | +1.54 | | 3p22.2 | participates in cell adhesion |
| **MCAM** | | +1.80 | | 11q23.3 | participates in cell adhesion |
| **MDM2** | | +1.00 | | 12q14.3-q15 | negative regulation of cell proliferation |
| **MET** | | +1.35 | | 7q31 | Proto-oncogene, promotes cell proliferation |
| **MGAT5** | | +1.67 | | 2q21 | promotes metastasis |
| **MMP11** | | +1.09 | | 22q11.23 | decomposes, protein hydrolysate and promotes metastasis |
| **MMP13** | | +1.20 | | 11q22.3 | decomposes, protein hydrolysate and promotes metastasis |
| **MTA1** | | +2.22 | | 14q32.3 | promotes metastasis |
| **MTSS1** | | +1.74 | | 8p22 | inhibits metastasis and proliferation |
| **MYCL1** | | +1.84 | | 1p34.2 | transcription factor, promotes proliferation |
| **NF2** | | +1.66 | | 22q12.2 | inhibits proliferation |
| **NME1** | | +2.12 | | 17q21.3 | negatively regulates proliferation and participates in cell adhesion |
| **NME2** | | +1.42 | | 16q13 | induces apoptosis |
| **NR4A3** | | +1.32 | | 9q22 | transcription factor, promotes proliferation |
| **PLAUR** | | +1.59 | | 19q13 | activator of plasminogen |
| **PTEN** | | +1.27 | | 10q23.3 | inhibits proliferation and metastasis |
| **RB1** | | +1.07 | | 13q14.2 | negative regulation of cell reproduction |
| **SSTR2** | | +1.17 | | 17q24 | inhibits proliferation |
| **TGFB1** | | +1.45 | | 19q13.1 | inhibits or promotes proliferation, promotes metastasis |
| **TIMP2** | | +3.50 | | 17q25 | inhibits metastasis |
| **TIMP3** | | +5.31 | | 22q12.3 | induces apoptosis, inhibits metastasis |
| **TSHR** | | +1.55 | | 14q31 | promotes proliferation |
| HPRT1 | +1.08 | | | Xq26.1 | nucleotide metabolism |
| **ACTB** | | +1.05 | | 7p15-p12 | ORM cytoskeleton |
| **Downregulated genes** | | | |  |  |
| **APC** | | -1.82 | | 5q21-q22 | cell adhesion, inhibits proliferation |
| **BRMS1** | | -1.45 | | 11q13-q13.2 | inhibits metastasis and tumor growth |
| **CCL7** | | -1.02 | | 16q13 | promotes invasion and migration |
| **CDH1** | | -1.60 | | 16q22.1 | inhibits tumor metastasis |
| **CDH11** | | -1.02 | | 16q22.1 | involves in the metastatic process |
| **CDH6** | | -1.34 | | 5p15.1-p14 | osteosis, cell adhesion |
| **CDKN2A** | | -1.25 | | 9p21 | negative regulation of cell cycle |
| **CHD4** | | -1.46 | | 12p13 | chromatin assemble and modification |
| **CTNNA1** | | -1.63 | | 5q31 | participates in cell adhesion |
| **CTSK** | | -2.78 | | 1q21 | protein hydrolysate |
| **CXCL12** | | -1.02 | | 10q11.1 | participates in cell adhesion |
| **CXCR4** | | -4.74 | | 2q21 | signal transduction, promotes invasion |
| **DENR** | | -1.33 | | 12q24.31 | promotes proliferation |
| **EPHB2** | | -1.45 | | 1p36.1-p35 | signal transduction, promotes invasion |
| **EWSR1** | | -1.86 | | 22q12. | transcription factor, promotes oncogenesis |
| **FAT1** | | -1.99 | | 4q35 | participates in cell adhesion |
| **GNRH1** | | -1.56 | | 8p21-p11.2 | inhibits cell proliferation |
| **HGF** | | -1.02 | | 7q21.1 | participates in proteolysis, promotes proliferation |
| **HRAS** | | -1.32 | | 11p15.5 | promotes proliferation |
| **HTATIP2** | | -1.27 | | 11q13 | positively regulates transcription |
| **IGF1** | | -2.10 | | 12q22-q23 | promotes proliferation |
| **IL1B** | | -1.41 | | 2q14 | inhibits or promotes proliferation |
| **CXCR2** | | -1.02 | | 2q35 | signal transduction, promotes invasion |
| **KRAS** | | -1.16 | | 12p12.1 | cell signal transduction, proliferation |
| **METAP2** | | -1.26 | | 12q22 | protein hydrolysate and modification |
| **MMP10** | | -1.02 | | 11q22.3 | protein hydrolysate, promotes metastasis |
| **MMP2** | | -1.02 | | 16q13-q21 | decomposes, protein hydrolysate and promotes metastasis |
| **MMP3** | | -1.01 | | 11q22.3 | decomposes, protein hydrolysate and promotes metastasis |
| **MMP7** | | -1.02 | | 11q21-q22 | decomposes, protein hydrolysate and promotes metastasis |
| **MMP9** | | -1.21 | | 20q11.2-q13.1 | decomposes, protein hydrolysate and promotes metastasis |
| **MYC** | | -1.64 | | 8q24.12-24.13 | promotes proliferation |
| **NME4** | | -1.28 | | 16p13.3 | inhibits proliferation |
| **PNN** | | -1.31 | | 14q21.1 | inhibits proliferation |
| **RORB** | | -1.02 | | 9q22 | participates in regulate of transcription |
| **SET** | | -1.46 | | 9q34 | inhibits histone acetylation |
| **SMAD2** | | -1.55 | | 18q21.1 | cell signal transduction |
| **SMAD4** | | -1.15 | | 18q21.1 | cell signal transduction |
| **SRC** | | -1.01 | | 20q12-q13 | promotes proliferation |
| **SYK** | | -1.05 | | 9q22 | promotes proliferation |
| **TCF20** | | -1.04 | | 22q13.3 | transcription factor |
| **TIMP4** | | -1.02 | | 3p25 | inhibits metastasis |
| **TNFSF10** | | -5.34 | | 3q26 | induces apoptosis, inhibits proliferation |
| **TP53** | | -1.28 | | 17p13.1 | induces apoptosis and cell differentiation, inhibits proliferation |
| **TRPM1** | | -1.02 | | 15q13-q14 | calcium channels |
| **VEGFA** | | -1.40 | | 6p12 | promotes proliferation, metastasis; inhibits apoptosis |
| **B2M** | | -1.63 | | 15q21-q22.2 | immune response, MHC I receptor |
| **RPL13A** | | -1.46 | | 19q13.3 | protein metabolism, promotes proliferation |
| **GAPDH** | | -1.10 | | 12p13 | glycometabolism |
