# Supplementary material for: The Role of EZH2 in the Regulation of the Activity of Matrix Metalloproteinases in Prostate Cancer Cells
Source: PLoS One. 2012 Jan 17;7(1):e30393. doi: 10.1371/journal.pone.0030393 (PMC3260297; doi:10.1371/journal.pone.0030393)
Supplement: Table S2 — List of genes differentially expressed in PC3 cells after EZH2 knockdown using a Human Tumor Metastasis Real-time PCR Array. (DOC) [file pone.0030393.s003.doc]

**Table S2. List of genes differentially expressed in PC3 cells after EZH2 knockdown using a Human Tumor Metastasis Real-time PCR Array**

| ***Gene*** | ***Fold change*** | ***Location*** | ***Function*** |
| --- | --- | --- | --- |
| **Upregulated genes** | |  |  |
| **BRMS1** | +1.27 | 11q13-q13.2 | inhibits metastasis and tumor growth |
| **CCL7** | +4.66 | 16q13 | promotes invasion and migration |
| **CD44** | +1.57 | 11p13 | cell adhesion and stroma attachment |
| **CHD4** | +1.66 | 12p13 | chromatin assemble and modification |
| **COL4A2** | +2.04 | 13q34 | the component of extracellular matrix |
| **CST7** | +1.33 | 20p11.21 | inhibits cysteine proteinase |
| **CTSL1** | +1.75 | 9q21-q22 | protein hydrolysate |
| **CXCR4** | +5.70 | 2q21 | signal transduction, promotes invasion |
| **EPHB2** | +2.77 | 1p36.1-p35 | signal transduction, promotes invasion |
| **ETV4** | +1.01 | 17q21 | transcription factor, promotes proliferation |
| **FGFR4** | +3.10 | 5q35.1 | promotes invasion |
| **FXYD5** | +1.28 | 19q12-q13.1 | negative regulation of cell adhesion |
| **KISS1R** | +1.02 | 19p13.3 | suppresses metastasis |
| **HGF** | +1.05 | 7q21.1 | participates in proteolysis, promotes proliferation |
| **HPSE** | +2.43 | 4q21.3 | hydrolyses protein |
| **HRAS** | +1.10 | 11p15.5 | promotes proliferation |
| **HTATIP2** | +1.24 | 11q13 | positively regulates transcription |
| **IL1B** | +4.17 | 2q14 | inhibits or promotes proliferation |
| **MCAM** | +1.24 | 11q23.3 | participates in cell adhesion |
| **MMP11** | +3.23 | 22q11.23 | decomposes, protein hydrolysate and promotes metastasis |
| **MMP2** | +4.03 | 16q13-q21 | decomposes, protein hydrolysate and promotes metastasis |
| **MMP3** | +1.05 | 11q22.3 | decomposes, protein hydrolysate and promotes metastasis |
| **MMP9** | +1.14 | 20q11.2-q13.1 | decomposes, protein hydrolysate and promotes metastasis |
| **MTSS1** | +4.03 | 8p22 | inhibits metastasis and proliferation |
| **MYC** | +1.25 | 8q24.12-24.13 | promotes proliferation |
| **NF2** | +2.91 | 22q12.2 | inhibits proliferation |
| **NME1** | +1.99 | 17q21.3 | negatively regulates proliferation and participates in cell adhesion |
| **NME2** | +1.14 | 16q13 | induces apoptosis |
| **NME4** | +2.45 | 16p13.3 | inhibits proliferation |
| **PLAUR** | +1.84 | 19q13 | activator of plasminogen |
| **RORB** | +1.44 | 9q22 | participates in regulate of transcription |
| **SRC** | +1.43 | 20q12-q13 | promotes proliferation |
| **SYK** | +1.01 | 9q22 | promotes proliferation |
| **TGF-B1** | +1.06 | 19q13.1 | inhibits or promotes proliferation, promotes metastasis |
| **TIMP2** | +2.08 | 17q25 | inhibits metastasis |
| **TIMP3** | +2.83 | 22q12.3 | induces apoptosis, inhibits metastasis |
| **TSHR** | +2.57 | 14q31 | promotes proliferation |
| **B2M** | +1.03 | 15q21-q22.2 | immune response, MHC I receptor |
| **HPRT1** | +1.04 | Xq26.1 | nucleotide metabolism |
| **Downregulated genes** | |  |  |
| **APC** | -1.56 | 5q21-q22 | cell adhesion, inhibits proliferation |
| **CDH1** | -1.28 | 16q22.1 | inhibits tumor metastasis |
| **CDH11** | -1.40 | 16q22.1 | involves in the metastatic process |
| **CDH6** | -3.73 | 5p15.1-p14 | osteosis, cell adhesion |
| **CDKN2A** | -1.75 | 9p21 | negative regulation of cell cycle |
| **CTBP1** | -1.30 | 4p16 | inhibits cell proliferation |
| **CTNNA1** | -1.58 | 5q31 | participates in cell adhesion |
| **CTSK** | -2.87 | 1q21 | protein hydrolysate |
| **CXCL12** | -6.19 | 10q11.1 | participates in cell adhesion |
| **DENR** | -1.46 | 12q24.31 | promotes proliferation |
| **EWSR1** | -1.11 | 22q12. | transcription factor, promotes oncogenesis |
| **FAT1** | -1.33 | 4q35 | participates in cell adhesion |
| **FLT4** | -1.07 | 17q21 | transcription factor, promotes proliferation |
| **FN1** | -1.74 | 2q34 | participates in cell adhesion |
| **GNRH1** | -1.89 | 8p21-p11.2 | inhibits cell proliferation |
| **IGF1** | -2.40 | 12q22-q23 | promotes proliferation |
| **IL18** | -1.20 | 11q22.2-q22.3 | promotes cell proliferation |
| **CXCR2** | -1.26 | 2q35 | signal transduction, promotes invasion |
| **ITGA7** | -2.22 | 12q13 | participates in cell adhesion |
| **ITGB3** | -1.67 | 17q21.32 | participates in cell adhesion |
| **CD82** | -1.38 | 11p11.2 | metastasis suppressor |
| **KISS1** | -2.58 | 1q32 | suppresses metastasis |
| **KRAS** | -2.53 | 12p12.1 | cell signal transduction, proliferation |
| **RPSA** | -1.21 | 3p22.2 | participates in cell adhesion |
| **MDM2** | -3.05 | 12q14.3-q15 | negative regulation of cell proliferation |
| **MET** | -1.33 | 7q31 | Proto-oncogene, promotes cell proliferation |
| **METAP2** | -2.13 | 12q22 | protein hydrolysate and modification |
| **MGAT5** | -1.05 | 2q21 | promotes metastasis |
| **MMP10** | -1.92 | 11q22.3 | protein hydrolysate, promotes metastasis |
| **MMP13** | -4.03 | 11q22.3 | decomposes, protein hydrolysate and promotes metastasis |
| **MMP7** | -3.73 | 11q21-q22 | decomposes, protein hydrolysate and promotes metastasis |
| **MTA1** | -1.04 | 14q32.3 | promotes metastasis |
| **MYCL1** | -2.07 | 1p34.2 | transcription factor, promotes proliferation |
| **NR4A3** | -2.55 | 9q22 | transcription factor, promotes proliferation |
| **PNN** | -2.13 | 14q21.1 | inhibits proliferation |
| **PTEN** | -1.15 | 10q23.3 | inhibits proliferation and metastasis |
| **RB1** | -3.34 | 13q14.2 | negative regulation of cell reproduction |
| **SET** | -1.47 | 9q34 | inhibits histone acetylation |
| **SMAD2** | -4.06 | 18q21.1 | cell signal transduction |
| **SMAD4** | -1.57 | 18q21.1 | cell signal transduction |
| **SSTR2** | -1.12 | 17q24 | inhibits proliferation |
| **TCF20** | -1.55 | 22q13.3 | transcription factor |
| **TIMP4** | -1.74 | 3p25 | inhibits metastasis |
| **TNFSF10** | -1.58 | 3q26 | induces apoptosis, inhibits proliferation |
| **TP53** | -1.06 | 17p13.1 | induces apoptosis and cell differentiation, inhibits proliferation |
| **TRPM1** | -3.97 | 15q13-q14 | calcium channels |
| **VEGFA** | -1.60 | 6p12 | promotes proliferation, metastasis; inhibits apoptosis |
| **GAPDH** | -1.02 | 12p13 | glycometabolism |
| **ACTB** | -1.10 | 7p15-p12 | ORM cytoskeleton |
| **RPL13A** | -1.03 | 19q13.3 | protein metabolism, promotes proliferation |
