# Supplementary material for: The Role of EZH2 in the Regulation of the Activity of Matrix Metalloproteinases in Prostate Cancer Cells
Source: PLoS One. 2012 Jan 17;7(1):e30393. doi: 10.1371/journal.pone.0030393 (PMC3260297; doi:10.1371/journal.pone.0030393)
Supplement: Table S3 — Primers for RT-PCR and ChIP analyses. (DOC) [file pone.0030393.s004.doc]

**Table S3. Primers for RT-PCR and ChIP analyses**

| ***Gene*** | ***Primes Sequence*** |
| --- | --- |
| **For RT-PCR** |  |
| ***EZH2*** | 5'- GCCAGACTGGGAAGAAATCTG -3' 5'- TGTGTTGGAAAATCCAAGTCA -3' |
| ***TIMP1*** | 5'- TTCCACAGGTCCCACAAC-3' 5'- CGTCCACAAGCAATGAGT-3' |
| ***TIMP2*** | 5'- CTGGACGTTGGAGGAAAGAA-3'  5'- GTCGAGAAACTCCTGCTTGG -3' |
| ***TIMP3*** | 5'- CTACCTGCCTTGCTTTGTGAC-3'  5'- ATCCTCGGTACCAGCTGCAG-3' |
| ***TIMP4*** | 5'- GCTGGGTGAGGCATGCAGCT-3'  5'- CAGGGTCTGCACTGGCCGGA-3' |
| ***GAPDH*** | 5'- TAGAAAAACCTGCCAAATATGATG-3'  5'- CTGTAGCCAAATTCGTTGTCATAC-3' |
| **For ChIP** |  |
| ***TIMP2 (set 1)*** | 5'- AGCCAGGCTGGTCTCAAACT-3' 5'- CAGTCCAGCTAGGGAGAGGG-3' |
| ***TIMP2 (set 2)*** | 5'- TCATATGCCTGGGTCTTTCC-3' 5'- GGGGGTGTGGTTACTGTGAA-3' |
| ***TIMP2 (set 3)*** | 5'- GTTTCTCAATAGGCCACCCG-3' 5'-TTCCCCTTCAGCTCGACTCT-3' |
| ***TIMP3 (set 1)*** | 5'- GGCTGGGCACTGTACAAAGA -3'  5'- AAGGAACTTGCCCATTTTCA -3' |
| ***TIMP3 (set2)*** | 5'- GCTAGAGTGCAG TGGCATGA -3'  5'- ACGAGGTCAGGAGATCGAGA -3' |
| ***TIMP3 (set3)*** | 5'- GTGGTTGGGGAAGCAATAGA -3'  5'- CTCCTTTTTCCCCTTTGAGC -3' |
| ***TIMP3 (set4)*** | 5'- TCTTCGGCCTCTGCTGTCCCA -3'  5'- GGTGGCCAGCCAGGAACTCG -3' |
| ***TIMP3 (set5)*** | 5'- ACAGGATGAAGCGGAAGAGA -3'  5'- TGTGGGTAGGAAAAGCAAGC -3' |
| ***TIMP3 (set6)*** | 5'- GCTTGCTTTTCCTACCCACA -3'  5'- CTGACAGAGCTCCACCCTTC -3' |
| ***TIMP3 (set7)*** | 5'- TGGTACCTCTCTCCTGTGGCT T -3'  5'- ACACAAAGCCCTTGCCCCCG -3' |
| ***TIMP3 (set8)*** | 5'- CGGGGGCAAGGGCTTTGTGT -3'  5'- TGGTGGAACCAGCGGGGGAA -3' |
| ***TIMP3 (set9)*** | 5'- TTCCCCCGCTGGTTCCACCA -3'  5'- CGTGCCCCTTCTCGTCTGCG -3' |
| ***TIMP3 (set10)*** | 5'- GCCAGGGCGCAGACGAGAAG -3'  5'- CGCCCCCTCAGACCAATGGC -3' |
| ***GAPDH*** | 5'- TACTAGCGGTTTTACGGGCG-3'  5'- TCGAACAGGAGGAGCAGAGAGCGA-3' |
